# Coinfection of Two Mycoviruses Confers Hypovirulence and Reduces the Production of Mycotoxin Alternariol in *Alternaria alternata* f. sp. *mali*

**DOI:** 10.3389/fmicb.2022.910712

**Published:** 2022-06-09

**Authors:** Bo Li, Yuhan Cao, Zixuan Ji, Jingyi Zhang, Xianglong Meng, Pengbo Dai, Tongle Hu, Shutong Wang, Keqiang Cao, Yanan Wang

**Affiliations:** State Key Laboratory of North China Crop Improvement and Regulation, College of Plant Protection, Hebei Agricultural University, Baoding, China

**Keywords:** mycovirus, mycotoxin, co-infection, *Alternaria alternata*, apple leaf blotch disease, hypovirulence

## Abstract

*Alternaria* leaf blotch caused by *Alternaria alternata* apple pathotype (*Alternaria mali*) is an important fungal disease that affects the production of apples worldwide. Mycoviruses harbored in plant pathogenic fungi can confer hypovirulence in their hosts and have attracted widespread attention as potential biocontrol tools. In this study, the coinfection of two mycoviruses, named *A. alternata* chrysovirus 1 strain QY2 (AaCV1-QY2) and *A. alternata* magoulivirus 1 (AaMV1), respectively, were isolated from *A. alternata* f. sp. *mali* strain QY21. Sequence analyses revealed that AaCV1-QY2 virus belonged to the genus *Betachrysovirus* and AaMV1 virus belonged to the genus *Magoulvirus.* These two mycoviruses were found to be associated with hypovirulence in *A. alternata*, among which AaCV1-QY2 might play a relatively leading role. Because the elimination of AaMV1 from the strain QY21 does not affect the hypovirulence trait, which indicates that the virus AaCV1-QY2 can independently induce slow growth and reduce host virulence. Moreover, the presence of viruses decreased the accumulation of the mycotoxin alternariol (AOH) in *A. alternata* strains. Intriguingly, AaCV1-QY2/AaMV1 mycoviruses can be horizontally transmitted to other *A. alternata* strains, and this coinfection can promote the interspecific transmission efficiency of AaCV1-QY2. To our knowledge, this study reports the first description of the member of *Chrysovirus* is related to hypovirulence in *Alternaria* spp. that facilitates the development of biocontrol measures of *A. mali* Roberts.

## Introduction

Mycoviruses (fungal viruses) are universally found in the main taxonomic groups of fungi, including phytopathogenic species. The majority of mycoviruses have double-stranded RNA (dsRNA) or positive-sense single-stranded RNA (+ssRNA) genomes, and they can or cannot have a capsid (Ghabrial et al., 2015). Due to the lack of external transmission pathways in most cases, mycoviruses are not typically infectious in the traditional sense (Yu et al., 2013). They are horizontal transmitted between strains mainly through hyphal anastomosis and vertically transmitted *via* spore production (García-Pedrajas et al., 2019). Most viral infections in fungi are commonly latent; however, some of them are capable of altering the phenotypic features and/or pathogenicity of fungal phytopathogenic hosts, that is, to say, conferring hypervirulence or hypovirulence of their hosts (Sun et al., 2020). From an agricultural perspective, innovative biocontrol measures to fungal diseases with the least negative impact on the environment and human health have attracted much more attention. In this context, the use of hypovirulent mycoviruses as control agents to protect plants from fungal diseases has become an up-and-coming biocontrol strategy.

During the last few decades, the most typical examples of the application of hypovirulent mycoviruses are that *Cryphonectria hypovirus* 1 (CHV1) is used to control chestnut blight caused by *Cryphonectria parasitica*, and is effectively commercialized in Europe (Martins et al., 2014). Because CHV1 infection causes phenotypic changes such as the suppression of sexual spore production, decreased pigmentation, and induces transmissible hypovirulence, which provides a foundation for biocontrol of chestnut blight. Except hypovirulence-assocated virus CHV1, which is a +ssRNA virus belonging to the family *Hypoviridae*, to date, several hypovirulence-inducing mycoviruses have been identified in the *Chrysoviridae* [*Botryosphaeria dothidea* Chrysovirus 1 (BdCV1)] (Ding et al., 2017), *Botourmiaviridae* [*Fusarium oxysporum* ourmia-like virus 1 (FoOuLV1)] (Zhao et al., 2020), *Partitiviridae* [*Colletotrichum liriopes* partitivirus 1 (ClPV1)] (Zhu et al., 2021), *Endornaviridae* [*Rhizoctonia solani* endornavirus 1 (RsEV1)] (Das et al., 2014), *Mitoviridae* [*Sclerotinia sclerotiorum* mitovirus 1 (SsMV1)] (Xu et al., 2015), *Reoviridae* [*Rosellinia necatrix* mycoreovirus 3 (RnMYRV-3)] (Yang et al., 2021), and *Genomoviridae* [*S. sclerotiorum* hypovirulence-associated DNA virus 1 (SsHADV-1)] (Yu et al., 2010) families form important phytopathogenic fungi. Moreover, the coinfection of mycoviruses is a very common phenomenon in nature. There are several interactions between co-infected mycoviruses, which variously regulate the phenotype and/or pathogenicity of the hosts according to the combination of virus partners, ranging from synergism to neutrality and antagonism (Thapa and Roossinck, 2019). For example, dual infection with Heterobasidion partitivirus 13 (HetPV13)-an1 and HetPV15-pa1 stably and drastically inhibited host growth, whereas the presence of co-infected viruses HetPV13-an1 and HetPV11-au1 had no or very little impact on their host (Kashif et al., 2019). The recognition that mycoviruses have the ability to induce hypovirulence in their hosts, which elicited great interest in the identification and characterization of viruses from phytopathogenic fungi because of their potential for controlling fungal diseases.

*Alternaria* species are very successful pathogenic genera that cause a wide range of plant diseases (Thomma, 2003). Approximately 300 species of the genus Alternaria have been identified worldwide, including *Alternaria tenuifolia*, *A. tenuifolia*, and *Alternaria solani*, of which *A. alternata* infects nearly 100 plant species. Some *A. alternata* strains even produce non-host-specific toxins (nHSTs) that induce phytotoxic effects on a broad range of plant species, generally as virulence factors, aggravating the severity of disease symptoms (Meena et al., 2017). *A. alternata* f. sp. *mali* is the forma specialis of *A. alternata* that infects apple (*Malus pumila Mill*), causing severe leaf blotch disease all over the world (Fontaine et al., 2021). Considering that *Alternaria* blotch disease has become a serious threat to apple production in China, it is necessary to explore hypovirulence-associated mycoviruses that may be used as virocontrol agents against *A. alternata* f. sp. *mali*.

To date, many mycoviruses infecting different *A. alternata* strains have been reported; however, few of them have been amply proven to possess hypovirulent effects on their hosts. The first mycovirus in *A. alternata* was discovered in 1988 with dsRNAs associated with virus-like particles in six isolates that produce tentoxin (Shepherd, 1988). To our knowledge, mycoviruses from more than six families have been found to be associated with *A. alternata*. These include multiple dsRNA mycoviruses *A. alternata* partitivirus 1 (AtPV1) (da Silva Xavier et al., 2018), *A. alternata* polymycovirus 1 (AaPmV1) (Ma et al., 2022), *A. alternata* victorivirus 1 (AalVV1) (Jamal et al., 2019), *A. alternata* virus 1 (AAV1) (Wu et al., 2021), *A. alternata* chrysovirus 1 (AaCV1) (Aoki et al., 2009; Okada et al., 2018), and *A. alternata* botybirnavirus 1 (AaBRV1) (Ma et al., 2019), classified as the families *Partitiviridae*, *Polymycoviridae*, *Totiviridae*, *Alternaviridae*, *Chrysoviridae*, and unassigned, respectively. Among them, AaCV1 infection has been shown to impair host growth in phenotype, but has been shown to enhance host virulence in pathogenicity by increasing the production of AK toxins during spore germination (Okada et al., 2018). Moreover, a novel ssRNA mycovirus *A. alternata* magoulivirus 1 (AaMV1), belonging to the family *Hypoviridae*, was identified from an apple orchard in China. And AaHV1 is the first mycovirus reported to slow the growth and attenuate the pathogenicity of *Alternaria* spp. (Li H. et al., 2019). Hypovirulence-associated mycovirus infection might be considered one of the meaningful mechanisms of natural decline of fungal diseases. However, in comparison with plant and animal viruses, the molecular characterization of mycoviruses and the virulence attenuation mechanisms of their host are still poorly understood.

In this study, we screened the dsRNAs in *A. alternata* f. sp. *mali* strain QY-2 isolated from an orchard in North China, which cause apple leaf blotch disease, and two hypovirulence-inducing mycoviruses, designated as *A. alternata* chrysovirus 1 strain QY2 (AaCV1-QY2) and *A. alternata* ourmia-like virus 1 (AaMV1) was identified. Meanwhile, we performed genome characterization to clarify the molecular properties of AaMV1 and AaCV1-QY2, investigate their influence on phenotypic alteration, pathogenicity, transmission efficiency, and nHST production in *A. alternata*. We also preliminarily explored the interplay between these two mycoviruses in the induction of hypovirulence during coinfection in the laboratory. This is the first description of the coinfection of mycoviruses associated with impaired growth and attenuation of virulence in *A. alternata*.

## Materials and Methods

### Fungal Isolates and Culturing Conditions

*Alternaria alternata* f. sp. *mali* strain QY-2 was isolated from an *Alternaria* blotch-diseased leaf of *Malus domestica* cv. Tengmu 1 in Hebei province of China in 2018. All collected and derived strains were cultured on potato dextrose agar (PDA) plate at 25°C in the dark. The hypovirulence strain QY21 is a single-conidium isolate progeny of QY-2. Mycelial plugs and asexual spores were preserved in glycerol (15%) at −80°C in the Lab of Plant Disease Epidemiology and IPM of Hebei Agricultural University (AUH), Baoding, Hebei province, China, all of which were verified by Koch’s rule and identified by sequencing of the translation elongation factor-1 α (*TEF-1*α) gene.

### RNA Extraction and Purification

The 5 mm mycelial plug of the strain QY21 was cultured on PDA medium overlaid with sterile cellophane for 4 days, then fungal mycelia were harvested and ground in liquid nitrogen. Viral dsRNA was extracted from 400 mg of mycelia or virus particles using cellulose (Sigma-Aldrich, St. Louis, MO, United States) column chromatography as previously described (Yang et al., 2021). Separated dsRNA samples digested with RNase-free DNase I and S1 nuclease were analyzed by electrophoresis in an agarose gel (1% wt/vol) at 80 V and visualized by staining with ethidium bromide under 350 nm ultraviolet (UV) illumination. Total RNA was isolated form 200 mg of fresh mycelia or Begonia (*Malus micromalus*) leaf using the TRIzol reagent (Newbio Industry, Wuhan, China) as described by the manufacturer. Total RNA quality was evaluated by agarose gel electrophoresis.

### cDNA Cloning and Sequencing

Virome sequencing was performed on the Illumina HiSeq 2000 sequencing platform by SHBIO Biotechnology Corporation (Shanghai, China). Total RNA of the strain QY21 was used as a template for deep sequencing analysis. Preparation of the cDNA library was performed with TruSeq RNA Sample Prep kit v2 (Illumina, San Diego, CA, United States). Raw reads were cleaned by removing adapter sequences, and low quality reads were trimmed with FASTP version 1.5.6 before assembly. All clean reads were assemble *de novo* using MEGAHIT version 1.0 (Li et al., 2016). The assembled contigs were classified based on viral sequence identities by BLASTX searches with the cutoff^1^ of *E* ≤ 1*e*^–5^. To obtain the complete sequence of mycoviruses, rapid RNA ligase-mediated amplification of cDNA ends (RACE) was performed using the SMART TM RACE cDNA Amplification kit (Takara, Dalian, China) according to the manufacturer’s protocol.

### Sequence and Phylogenetic Analyses

Sequence assembly of each region of genome was performed with the SeqMan software v7.1.0 (DNASTAR, Inc., Madison, WI, United States). The open reading frames (ORFs) and conserved domains of the mycoviruses were predicted from the National Center for Biotechnology Information (NCBI) website, and their homologous amino acid (AA) sequences were searched using BLASTp programs, respectively. Multiple sequence alignment was performed using the CLUSTAL X v2.0 (Larkin et al., 2007). A maximum likelihood (ML) phylogenetic tree was constructed based on AA alignments using MEGA X with 1,000 bootstrap replicates (Kumar et al., 2018). The virus reference sequence used for comparative analyses in this study was obtained from the NCBI database.

### Reverse Transcription-Polymerase Chain Reaction Detection

For reverse transcription-polymerase chain reaction (RT-PCR) detection of the different mycoviruses in fungal strains, first-strand cDNA was synthesized using the TransScript One-Step gDNA Removal and cDNA Synthesis SuperMix kit (TransGen Biotech, Shanghai, China) using total RNA as template. Then, the segmental sequences were amplified using Taq DNA polymerase (TaKaRa, Dalian, China) with the specific primers (AaCV1-QY2f and AaCV1-QY2r for ORF1 of AaCV1-QY2; AaMV1f and AaMV1r for ORF6 of AaMV1) to identify the presence of the corresponding mycoviruses. All primers used in this study are listed in Supplementary Table 1.

### Purification of Virus Particles

Mycoviruses-infected QY21 strain was used for virus particle purification. Approximately 20 g of mycelia cultured on PDB for 7 days were harvested and ground into fine power in liquid nitrogen. The obtained powder was homogenized with 0.1 M phosphate buffer (pH 7.0) containing 0.1% β-mercaptoethanol and shaken at 150 rpm for 30 min at 4°C, and then centrifuged two times at 10,000 × *g* for 15 min to remove cell debris. The resulting supernatant was further ultracentrifuged at 100,000 × *g* for 1 h (Optima LE-80K, Beckman Coulter, Inc., United States) to collect pellets. The precipitates were resuspended in 0.5 ml of PB buffer. Followed by centrifugation at 10,000 × *g* for 1 min, the final supernatants were overlaid and ultracentrifuged at 70,000 × *g* for 3 h in sucrose density gradients (10–60%). Virus particle fractions were further centrifuged at 100,000 × *g* for 3 h, and the resulting pellets were re-suspended in 50 μl of 0.05 M PB buffer. The purified virus particle was visualized with the transmission electron microscope (TEM) Hitachi H-7650 (Tokyo, Japan) after staining with 2% (wt/vol) phosphotungstic acid solution (pH 7.4).

### Elimination of the Mycovirus From the Strain QY21

We performed cycloheximide treatment to eliminate the virus from the strain QY21 as previously described with minor modifications (Shah et al., 2020). Briefly, 0.2-mm hyphal tips or a single spore of the strain QY21 was transferred to PDA medium containing two different chemicals 100 μg/ml ribavirin and 100 μg/ml cycloheximide, and cultured at 25°C for 5 days in the dark. After three –to five successive treatments, hyphal tips were subcultured on PDA at 25°C for 7 days. After successive transfer culture three to five times on cycloheximide-treated PDA medium, the hyphal tips were individually subcultured on cycloheximide-free PDA at 25°C for another 5 days. Finally, the presence of the virus in the regenerated fungal strains was detected by RT-PCR.

### Extraction and Quantification of Mycotoxins

After removal of the mycelium cultured on PDA plates, 1 g of agar plugs were cut from the center of 7-day-old fungal colonies and placed in a 5-ml microcentrifuge tube. Extraction solvent [ethyl acetate/dichloromethane/methanol (3: 2: 1, v/v/v)] containing 2% formic acid was added to each tube, and the plugs were extracted by ultrasonic treatment for 60 min. Then the supernatant was transferred to a clean tube after centrifugation at 4,000 × g for 2 min and freeze-drying the samples for 12–24 h to remove all water. Thereafter, the samples were redissolved in 500 μl of methanol-water (50:50 v/v) with 1% formic acid and filtered through a 0.22-μm pore size nylon membrane (RephiLe Bioscience, Philadelphia, PA, United States). Mycotoxins in the extract were then quantitatively analyzed by high-performance liquid chromatography (HPLC) Agilent 1290 system (CA, United States) using an Agilent Eclipse Plus C18 (50 nm × 2.1 mm × 1.8 μm).

The sample injection volume was 2 μl with a flow rate of 0.25 ml/min at 40°C. The mobile phase was composed of phases A (water) and B (methanol), both with 1% formic acid. The elution program was set as follows: 10% B (initial), 10–90% B (0–6 min), 90% B (6–7 min), 90–10% B (7–7.1 min), 10% B (7.1–9 min), and held for further 3 min for re-equilibration (total run time 12 min). The mycotoxin alternariol (AOH) stock standard was diluted with methanol-water (50:50 v/v) solution containing 1% formic acid to six concentrations (2.5, 5, 10, 50, and 100 μg/ml) to construct a calibration curve, so as to determine the concentration of mycotoxin in the samples. The toxigenic potential of the virus-free and virus-infected strains was evaluated by testing the pathogenicity of 10 μl of mycotoxins extracted from the different strains inoculated in Begonia leaves.

### Horizontal Transmission Assay

The co-culture experiment was chosen to evaluate the potential horizontal transmission of viruses harbored in *Alternaria* strains. Virus-carrying strains QY21 or QY21-C1 were used as virus donors; the virus-free isogenic strain QY21-C2 or other 99 virus-free allogenetic strains were used as virus recipients, respectively. First, a mycelial plug of the fungal strains QY21 or QY21-C1, respectively, was co-cultured for 14 days on a fresh PDA plate with mycelial plug of each recipient, which was kept close to each other (10–15 mm). Then, the mycelial plugs, which were close to the recipient strain but far away from the strain QY21, are transferred to a new PDA plate and cultured in the dark at 25°C for 7 days. Virus transport in these derivative strains was evaluated based on visual observation of distinctive mycovirus-associated colony morphologies and virus detection by RT-PCR with virus-specific primers.

To determine the transmission trait of the mycovirus of virus-infected strain QY21 to Begonia leaves. Plugs of virus-carrying and virus-free fungal strains were inoculated onto Begonia leaves, respectively. After 7 days of culture, total RNA was extracted from leaves (0, 1, and 2 cm away from the diseased spot or inoculated site). The presence of the virus in leaves was confirmed by RT-PCR detection.

### Vertical Transmission Assay

Conidia collected from the cultures of the infected strains QY21 and QY21 were used to prepare the subculture of single spore to analyze mycovirus vertical transmission rates. A total of 100 single-spore cultures were carried out for each virus-carrying strain. The number of single-spore isolates containing the virus AaCV1-QY2 was recorded. The presence of the virus in these isolates was confirmed by RT-PCR detection.

### Effects of the Mycovirus on Host Biological Properties

To assess morphology and growth rates, 4-day-old mycelial plugs of the strains QY21, QY21-C1, and QY21-C2 were transferred to a PDA plate and cultured at 25°C for 3–7 days in the dark. The morphology of colonies was photographed, and the morphology of hyphae and spores was observed with a light microscope. And the diameters of each colony were measured using the crisscross method at 7 days to calculate mycelial growth rates, with each strain having five replicates.

The potential effects of mycoviruses on spore production and germination were investigated using the following method. Fresh 5 mm fresh mycelial plugs of each strain were inoculated on PDA medium under near-UV light (12 h light/12 h dark) for 7 days. Spores were collected by flushing the culture with sterile distilled water containing 0.05% (v/v) Tween and residual mycelia were removed by filtration through a glass fiber filter. The spore production of mycelia per square centimeter was measured with a hemocytometer. For the spore germination assay, the concentration of spore suspensions of each strain was adjusted to 1 × 10^5^ spores/ml with sterile distilled water. The prepared spore suspension was dropped onto a concave glass slide and placed on a culture dish containing wet filter paper for 18 h. The germination rate of spores was calculated by counting the number of germinated spores. Spores were considered to have germinated when bud tube length is equal to or greater than the diameter of spores. Approximately 100 spores of *A. alternata* per strain were observed within each replicate, and all experiments were repeated three times.

### Effects of the Mycovirus on Host Virulence

For virulence assessment, mycelial plugs or mycotoxin of each strain were inoculated on detached apple fruit and Begonia leaves. Diameters of lesions developed after 7 days of inoculation were photographed and measured. All tests were maintained at 25°C in a humid container. Each of the above tests was conducted three times, and each strain had five replicates.

### Statistical Analyses

Statistical analyses were performed using a one-way analysis of variance (ANOVA) followed by *post hoc t*-tests to determine significant differences at a significant level of *p* < 0.05 with SPSS 22.0 software. Unless otherwise stated, all experiments were repeated at least three times independently.

## Results

### Viruses in Pathogenic *A. alternata* Strain QY-2

**FIGURE 1:**
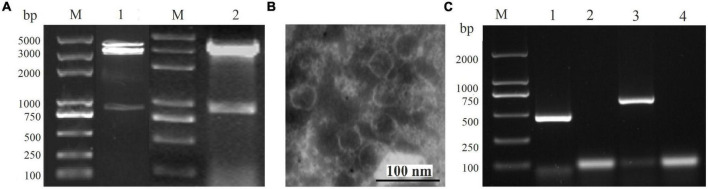
Infection of *Alternaria alternata* chrysovirus 1 strain QY2 (AaCV1-QY2) and *A. alternata* magoulivirus 1 (AaMV1) in *A. alternata* QY21 strain. **(A)** Agarose gel electrophoresis analysis of the double-stranded RNA (dsRNA) extracted from the mycelium (lane 1) and virus particles (line 2), lane M is for markers. **(B)** Electron micrograph of purified particles of mycoviruses extracted from the strain QY21. The scale bar denotes 100 nm. **(C)** AaCV1-QY2 (lane 1) and AaMV1 (lane 3) in the strain QY21 were detected by reverse transcription-polymerase chain reaction (RT-PCR) using specific primers AaCV1-QY2f/r or AaMV1f/r directed to the RNA-dependent RNA polymerase (RdRp) sequence of mycoviruses AaCV1-QY2 and AaMV1, respectively. Lane M is for markers; lanes 2 and 4 represent the negative controls (NCs).

The dsRNA was extracted from the virus particles of *A. alternata* strain QY21, and it was treated with DNase I and S1 nuclease before 1% agarose gel. Multiple dsRNAs were detected, especially three distinct dsRNA bands with the size of approximately 0.8, 3.0, and 3.5 kbp, respectively, indicating the presence of viral infection in the strain QY21 (Figure 1A). The next-generation sequencing (NGS) result showed the presence of six virus-like contigs derived from the different dsRNA molecules of the isolate QY21. Those contigs were used for homology searches against the NCBI virus AA sequence database using BLASTx. We found that two novel mycoviruses associated with the members of *Betachrysovirus* in the family *Chrysoviridae* and the members of *Magoulvirus* in the family *Botourmiaviridae*, respectively, were harbored in the causal agent of Apple *Alternaria* Blotch. Virus particles with a size of around 30–40 nm in diameter were purified from the mycelium of the QY-2 isolate (Figure 1B), and the extraction of dsRNA from the purified virus particles and RT-PCR with primers corresponding to the two identified viruses confirmed the genomic profile previously detected in the initial screening (Figures 1A,C).

### Molecular Characterization of Mycovirus Associated With the Strain QY21

**FIGURE 2:**
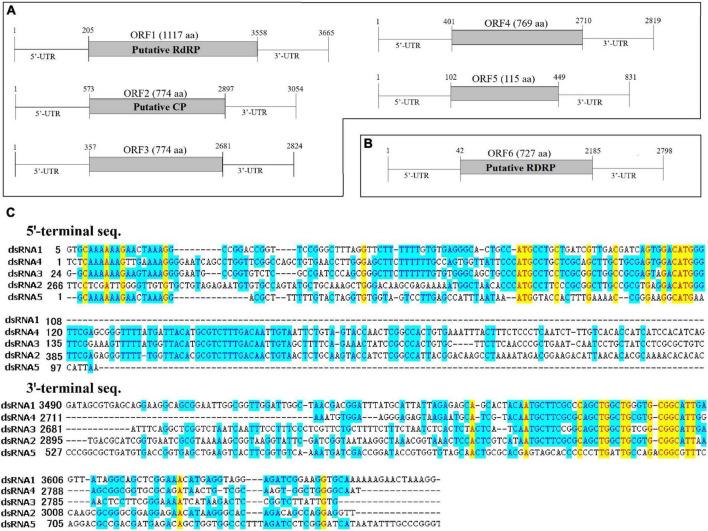
The genome organization and the terminal structure of AaCV1-QY2 and AaMV1. **(A,B)** A schematic diagram of the genome organization of AaCV1-QY2 (A) and AaMV1 **(B)**. The boxes and lines represent the open reading frames (ORFs) and untranslated regions (UTRs), respectively. RdRp represents viral RNA-directed RNA-polymerase domain; CP represents coat protein. **(C)** Multiple sequence alignments of the 5’- and 3’-terminal regions of the coding strands of the five AaCV1-QY2 dsRNA segments.

The full-length cDNA sequences of the dsRNA segments were determined by the combination of RT-PCR with specific primers (Supplementary Table 1) and the rapid cDNA ends amplification (RLM-RACE) protocol. As a result, six complete genomic sequences from dsRNA1 to dsRNA6, 3,665, 3,054, 2,824, 2,819, 831, and 2,798 bp in length, respectively, were obtained. Each dsRNA strand encoded a single putative ORF on the positive strand of the genome (Figure 2A). The dsRNA1-6 were designated as ORF1, ORF2, ORF3, ORF4, ORF5, and ORF6, respectively. All dsRNA sequences of AaCV1-QY2 and AaMV1 were deposited in GenBank with accession numbers MK672910, MK672913, MK672912, MK672911, MK836314, and MW492539, respectively. The NGS raw data were uploaded to the NCBI database (SRA accession: PRJNA832420).

The putative genomic organization of AaMV1 and AaCV1-QY2 were shown in Figures 2A,B. BLASTp search against NCBI databases revealed that ORF1-encoded protein displayed the highest identity to the RNA-dependent RNA polymerase (RdRp) of AaCV1 (accession no: LC350277.1, identity: 93%). The length of ORF1 is 3,351 bp, which has the potential to encode 1,117 AA residues contained a putative RdRp domain, starting at position 205 nt with an AUG codon and terminating with a UAA codon at positions 3,556–3,558 bp (Figure 2A). ORF2-encoded protein showed the highest sequence similarity to the putative coat protein of AaCV1 (accession no: LC350278.1, identity: 91%). No known domains were predicted in ORF3, ORF4, or ORF5; however, ORF3-, ORF4-, and ORF5-encoded proteins showed the highest sequence identities with proteins deduced from dsRNA3, dsRNA4, and dsRNA5 of AaCV1, respectively. The 5′-untranslated regions (UTRs) are relatively conserved among the five RNA segments of AaCV1-QY2 (Figure 2C). With the exception of segment 4, the 5′-terminal sequences of each of the other segments of AaCV1-QY2 contain a highly conserved motif (5′-GCAAAAAAGxxxxAAAGG-3′), which is a typical motif for viruses in the family Chrysoviridae (cluster II). Meanwhile, the conserved motif 5′-CGGC(A/G)TT-3′ appears in 3′-UTRs of all five segments (Figure 2C), and similar sequence structures are found in 3′-termini region of reported chrysoviruses such as AaCV1-N18 and AaCV1-AT1. Thus, this virus containing five ORFs was designated as AaCV1-QY2. Likewise, the genome of another virus contained only one large ORF6 that initiated at position 43 bp and terminated at positions 2,182–2,184 bp, potentially encoding 727 AA residues from an AUG codon to a UAG codon (Figure 2B). OFR6-encoded protein showed the most closely related to the RdRp of *Cladosporium cladosporioides* ourmia-like virus 1 (CcOLV1, accession no: MK584838, identity: 90%), so it was temporary named as *A. alternata* ourmia-like virus 1 (AaMV1).

**FIGURE 3:**
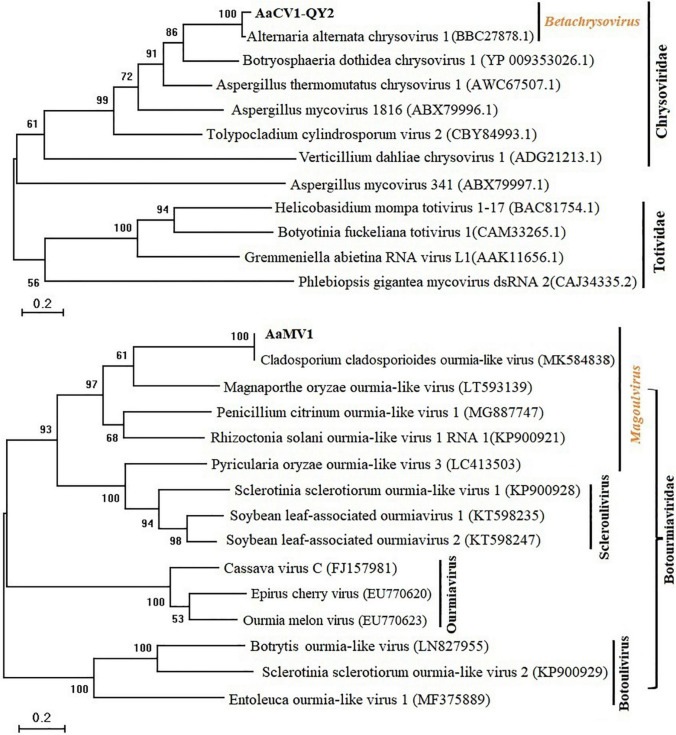
Maximum likelihood (ML) phylogenetic tree based on the RdRps of AaCV1-QY2 (Upper) or AaMV1 (Lower) and other related viruses. The phylogenetic tree was generated using the software MEGA X. The bootstrap values (%) were obtained with 1,000 replicates. The scale bar at the lower left represents a genetic distance of 0.2.

To clarify the evolutionary status of the AaCV1-QY2 or AaMV1, maximum likelihood phylogenetic analyses were performed based on the alignment of RdRp AA sequences of AaCV1-QY2 and AaMV1, respectively, with other related mycoviruses (Figure 3). RdRp-based phylogenetic tree showed that AaCV1-QY2 clustered together with AaCV1, suggesting that AaCV1-QY2 is a new strain of the genus *Betachrysovirus* in the family *Chrysoviridae* (Figure 3, top). In addition, phylogenetic analysis also indicated a close relationship of AaMV1 to CcOLV1, the member of the genus *Magoulvirus* in the family *Botourmiaviridae* (Figure 3, bottom). Combined with the above results, AaMV1 derived from *A. alternata* might belong to a new species of *Magoulvirus*.

### Effects of AaCV1-QY2/AaMV1 on the Biological Properties of *A. alternata*

**FIGURE 4:**
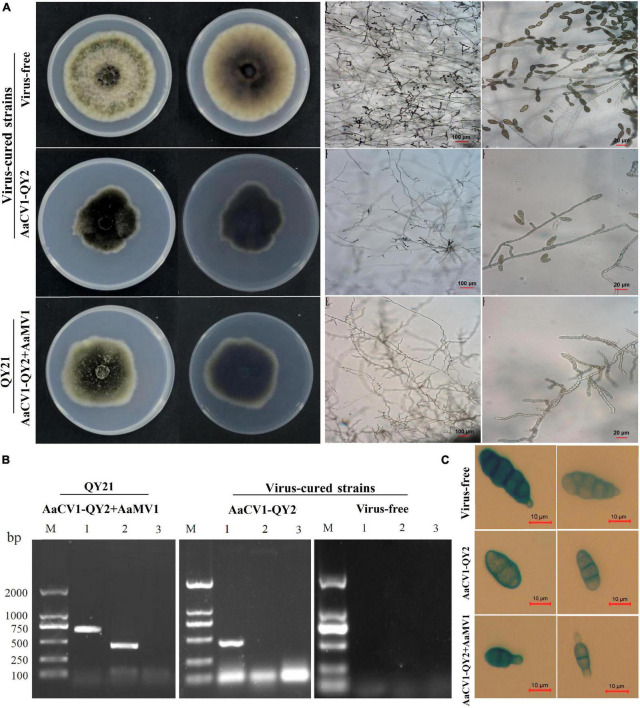
Virus elimination and biological properties of the co-infected and isogenic virus-cured *A. alternata* strains. The strain QY21 is doubly infected with AaCV1-QY2 and AaMV1; the strain QY21-C1 has eliminated the virus AaMV1 and retains the virus AaCV1-QY2; QY21-C2 strain has completely eliminated all viruses and is virus-free. **(A)** Colony and mycelial morphology of QY21 and virus-cured strains [potato dextrose agar (PDA), 4 days, 25°C]. **(B)** The dsRNA of AaCV1-QY2 and AaMV1 in the co-infected and virus-cured strains was detected by RT-PCR amplification. Lane M is for markers; lanes 1 and 2 show the results of PCR performed with AaCV1-QY2 and AaMV1 specific primers, respectively; lane 9 is for NC. **(C)** Spore morphology of strains (PDA, 4 days, 25°C).

To determine the effect of AaCV1-QY2 and AaMV1 on *A. alternata*, we attempted to cure the virus-infected strain of *A. alternata* by chemical treatment. Two types of virus-cured strains were obtained, namely, complete virus-free strain QY21-C2, and only AaCV1-QY2 virus-infected strain QY21-C1 (Figure 4B). In addition, we observed that AaCV1-QY2 was vertically transmitted through conidia with an efficiency of approximately 95% regardless of the presence of AaMV1 in the subculture strains. However, we did not succeed when we attempted to obtain a single AaMV1 virus-infected strain. This means that AaMV1 cannot be solely and stably accumulated in the cured strains after fungal subculture.

**FIGURE 5:**
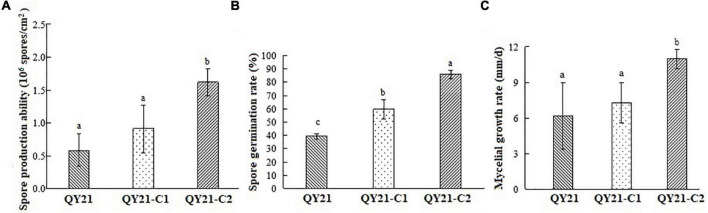
Biological comparison of the co-infected strain QY21 and the isogenic virus-cured strains QY21-C1 and QY21-C2. The strain QY21 is doubly infected with AaCV1-QY2 and AaMV1; the strain QY21-C1 has eliminated the virus AaMV1 and retains the virus AaCV1-QY2; QY21-C2 strain has completely eliminated all viruses and is virus-free. **(A)** The number of spores produced by the strains QY21, QY21-C1, and QY21-C2 (PDA, 7 days, 25°C). **(B)** Spore germination rate (PDA, 25°C). **(C)** Radial mycelial growth rate (PDA, 25°C). Different letters indicate a significant difference at *p* < 0.05 according to the *post hoc t*-tests.

The biological characteristics of the original strain QY21 and the two virus-cured strains QY21-C1 and QY21-C2 were compared, including colony and spore morphology, mycelial growth, and sporulation ability. The colony morphology of the virus-free strain QY21-C2 exhibited a round and significantly higher mycelium density than that of the virus-infected strains QY21 and QY21-C1, while the colony of the virus-infected strains had less and collapsed aerial mycelium and a larger area in black pigments (Figure 4A, left). Also, the mycelia of the strains QY21 and QY21-C1 formed an abnormal configuration, which showed irregular and shorter ramification and poor sporulation compared with the virus-free strain QY21-C2. Especially, the strain QY21-C1 displayed more and distorted ramification with some parts of the mycelia contracted and some parts swelled (Figure 4A, right). Moreover, we found that the spore length of AaCV1-QY2- and AaMV1-infected strain QY21 was shorter than that of the other two strains (Figure 4C), which explained that the spore production ability of the virus-infected strain QY21 (5.2 × 10^5^ spores/cm^2^) was significantly lower than that of the virus-free strain QY21-C2 (1.6 × 10^6^ spores/cm^2^) (Figure 5A).

In addition to the influence of virus infection on the morphology of fungal host, the mycelial growth and spore germination rate of virus-infected strains was significantly inhibited compared with the virus-free strain (Figures 5B,C). The result indicated that the spore germination rate of AaCV1-QY2- and AaMV1-co-infected strain QY21 was the lowest (39.3%), not only lower than that of the virus-free strain QY21-C2 (57.2%), but also statistically lower than that of the AaCV1-QY2-infected strain QY21-C1 (82.7%). These results suggested that AaMV1/AaCV1-QY2 infection probably reduced host strain virulence by inhibiting mycelial growth and spore production.

### Effects of AaCV1-QY2/AaMV1 on the Pathogenicity of *A. alternata*

**FIGURE 6:**
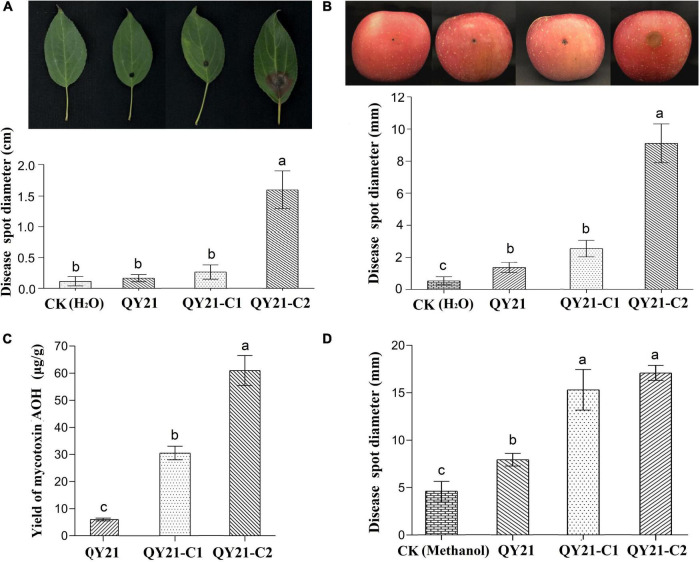
The virulence levels of *A. alternata* virus-infected strain QY21 and the isogenic virus-cured strains QY21-C1 and QY21-C2. **(A)** Disease spot diameter and representative images showing the lesions on *Malus begonia* leaf induced by *A. alternata* strains. **(B)** Disease spot diameter and representative images showing the lesions on apple fruits induced by *A. alternata* strains. The lesions were photographed at 5 days post-inoculation. **(C)** The yield of the mycotoxin alternariol (AOH) of different *A. alternata* strains. **(D)** Lesion lengths on the Begonia leaf induced by the mycotoxin AOH extracted from *A. alternata* strains. Different letters indicate a significant difference at *p* < 0.05 according to the *post hoc t*-tests.

To verify and evaluate the hypovirulence-inducing effect of the mycoviruses AaMV1/AaCV1-QY2 on the fungal host, virulence assays were performed on detached apple fruits and Begonia leaves. After 7 days of inoculation, the average diameter of diseased spots on Begonia leaves caused by the virus-infected strains QY21 (0.18 cm) and QY21-C1 (0.21 cm) were significantly smaller than that caused by the virus-free strain QY21-C2 (1.51 cm) (Figure 6A). Meanwhile, similar results were obtained in pathogenicity evaluation of the viruses on apple fruits. The average diameter of lesion areas in apple fruits inoculated with the virus-infected strains QY21 (1.78 mm) and QY-2-C1 (2.69 mm) decreased meaningfully compared with fruits inoculated with the virus-free strain QY21-C2 (9.15 mm) (Figure 6B). Taken together, these results indicated that AaMV1/AaCV1-QY2 might confer hypovirulence to its fungal host.

In this study, AaMV1/AaCV1-QY2 attenuated the nHST-producing ability of the host strain. According to the HPLC results, the virus-free strains QY21-C2 have the highest AOH toxin production, which is about two times as much as that of the AaCV1-QY2 virus-carrying strain QY21-C1 and approximately 10 times as much as that of the AaMV1 + AaCV1-QY2 virus-carrying strain QY21 (Figure 6C). Generally, the mycotoxin AOH production is positively correlated with the potential risks to human health and the pathogenicity of pathogenic fungi *A. alternata*. In this study, the virulence assay of toxins extracted from the three *A. alternata* strains in this study showed that total mycotoxins of the strains QY21-C1 and QY21-C2 had a remarkably stronger pathogenicity in Begonia leaves than in the strain QY21 (Figure 6D).

### Transmission and Colonization of AaMV1/AaCV1-QY2 in the Fungal Host and Plants

**FIGURE 7:**
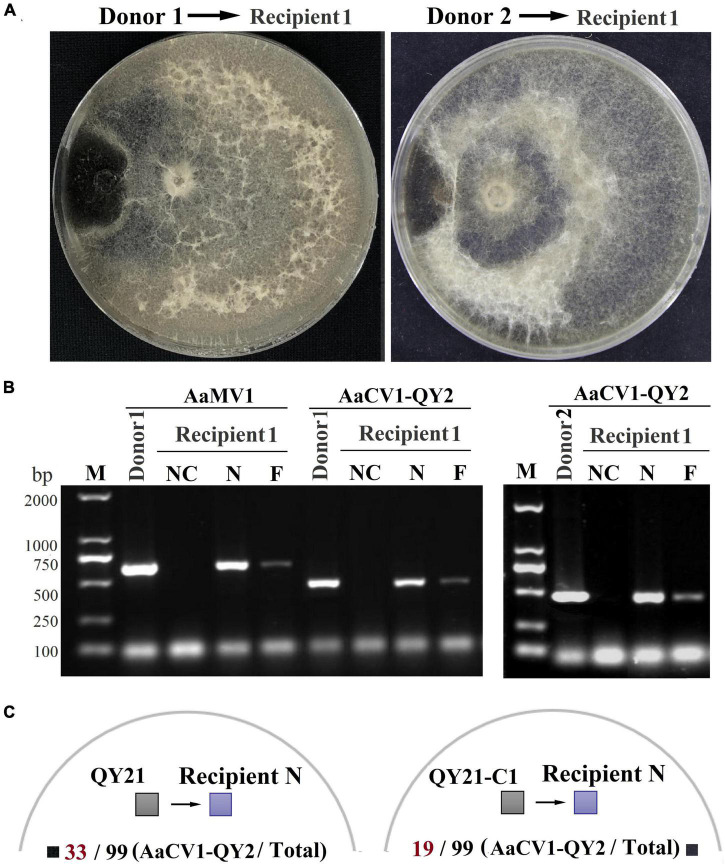
The efficiency of AaCV1-QY2 and AaMV1 horizontal transmission *via* hyphal anastomosis in *A. alternata*. The strain QY21 is doubly infected with AaCV1-QY2 and AaMV1; the strain QY21-C1 has eliminated the virus AaMV1 and retains the virus AaCV1-QY2; QY21-C2 strain has completely eliminated all viruses as virus-free. **(A)** Co-culture on PDA plates of the virus-infected strains QY21 (left) and QY21-C1 (right) as donors and the isogenic virus-free strain QY21-C2 as the recipient, respectively. After 2 weeks of hyphal contact, mycelial plugs were collected from two locations (N: near and F: far distance from the hyphal fusion areas) in the recipient side, transferred onto new PDA plates for dsRNA extraction; NC, negative control. **(B)** The detection of viral dsRNAs of AaCV1-QY2 or AaMV1 in recipient strains co-cultured with the AaCV1-QY2 + AaMV1-infected strain QY21 (left) and the AaCV1-QY2-infected strain QY21-C1 (right). **(C)** A schematic diagram of co-culture on PDA plates of the virus-infected strains QY21 (left) and QY21-C1 (right) as donors and the other 99 allogenetic virus-free strains collected from different orchards as recipients. The numbers below the schematic diagram indicate the number of samples with AaCV1-QY2 detected in the total number of samples.

The horizontal transmission ability of the mycovirus among single, double virus-infected, and virus-free strains was determined by co-culture and hyphal anastomosis (Figure 7A). The results revealed that the recipient strain QY21-C2 was successfully transfected with AaMV1 and/or AaCV1-QY2 obtained from the donor strains QY21 and QY21-C1, yielding the derived strains (recipients 1 and 2) that showed an obvious virus-infected phenotype in subculture (Supplementary Figure 2). The presence of AaMV1/AaCV1-QY2 was confirmed by RT-PCR with specific primers (Figure 7B). In addition, the colony morphology of the derived strains was similar to that of the original virus-infected strains QY21 or QY21-C1. The virulence assay showed that the hypovirulence traits were also successfully transmitted, and the symptoms of apple fruit or Begonia leaves inoculated with the derived strains were obviously alleviated relative to those inoculated with the virus-free strain, which appeared in the same way as the strains QY21 or QY21-C1 (Supplementary Figure 2). To further examine whether the coinfection condition affects the interspecific virus transmission ability of AaCV1-QY2, AaCV1-QY2, and AaMV1 + AaCV1-QY2-infected strains were co-cultured with the other 99 allogenetic virus-free strains (recipient N) collected from apple orchards. The results of co-culture assays showed that AaCV1-QY2 could effectively spread to the recipient side of heterologous fungi, regardless of the type of virus-infected strains used as donors. Also, the interspecific transmission efficiency of AaCV1-QY2 with double virus-infected strain as the donor is much higher than that with a single AaCV1-QY2 virus-infected strain as the donor (Figure 7C and Supplementary Figure 3), suggesting that coinfection with AaMV1 interferes with the efficient horizontal transmission of AaCV1-QY2 in *A. alternata*.

On the other hand, although Begonia is the host of *A. alternata* strain QY21, the virus harbored in QY21 cannot be transferred to Begonia leaves. According to our RT-PCR detection results, the viruses AaCV1-QY2/AaMV1 can only be detected in the lesion area, but not at 1 and 2 cm away from the lesion area (Supplementary Figure 1). Therefore, it is demonstrated that AaCV1-QY2/AaMV1 cannot colonize in the leaves of Begonia, which also indicates that the mycoviruses in this study are harmless to plants.

## Discussion

The identification of more hypovirulence-associated mycoviruses not only contributes to a better knowledge of viral variety and evolution, but also provides more resources with valuable biological potential (Sun et al., 2020). With the development of NGS technology, it is more feasible and convenient to detect and discover novel and meaningful viruses from fungi, plants, insects, and other environmental samples (Barba et al., 2013; Shahid et al., 2021). In this study, we identified two mycoviruses (AaMV1 and AaCV1-QY2) that co-infect an isolate of the plant pathogenic fungus *A. alternata* f. sp. *mali* strain QY21 *via* NGS sequencing. Of them, AaCV1-QY2 presents chrysovirus-like properties, with a genome consisting of five dsRNA segments between 1.0 and 3.5 kbp in length, encapsidated by isometric particles of approximately 30 nm in diameter. The virus AaCV1-QY2 clustered together with AaCV1 derived from *A. alternata* Japanese pear pathotype strain N18, the member of the genus *Betachrysovirus* in the family of *Chrysoviridae*. On the other hand, only one type of viral particle was detected in this study, presumably because AaMV1 was temporary assigned as ourmia-like mycovirus, which is one of the simplest viruses. Its genome usually consists of a (+)ssRNA with a size of 2.3–3.6 kb and includes a single ORF encoding RdRp. In addition, ourmia-like mycoviruses frequently lack coat protein and movement protein, hence the genomes are commonly found in the lipid vesicles of cells as an RNA/RdRp nucleoprotein complex rather than as virus particles (Wang et al., 2020). Therefore, the virus particles observed in this study may be formed by the virus AaCV1-QY2 (Figure 1B). This is also consistent with previous research findings: dsRNA mycoviruses are generally considered to have obvious virus particles, such as *Chrysoviridae*, *Megabirnaviridae*, *Partitivridae*, *Quadriviridae*, *Reoviridae*, and *Totiviridae*, whereas mycoviruses without obvious virus particles are considered as ssRNA viruses such as *Endornaviridae* and *Narnaviridae* (Ghabrial et al., 2015). To the best of our knowledge, this was the first report of two mycoviruses from different families co-infecting in *A. alternata* f. sp. *mali*.

In this study, we also found these viruses confer hypovirulence in *A. alternata*, the causal fungal pathogen of apple blotch disease. The virus-free *A. alternata* strain CY21-C2 induced the largest lesions on apple fruits and Begonia leaves, while *A. alternata* strains infected with AaCV1-QY2 or AaCV1-QY2 + AaMV1 exhibited significantly smaller lesions (Figures 6A,B). It suggests that single AaCV1-QY2 infection or double AaCV1-QY2/AaMV1 infection strongly reduces the virulence of *A. alternata*. Typically, hypovirulence-associated mycovirus infection can cause important physiological changes such as the malformation of morphological characters, the retardation of vegetative growth, and the inhibition of conidia production, thus affecting pathogenic behavior (Sharma et al., 2018; Li P. et al., 2019). Our observations also showed that the presence of the mycovirus AaCV1-QY2 alone or in combination with AaMV1 in *A. alternata* altered the host morphology, which might be one of the important factors in debilitating fungal host virulence. On the other hand, similar levels of hypovirulence were observed between *A. alternata* strains QY21 with double AaCV1-QY2 + AaMV1 infection and QY21-C1 with single AaCV1-QY2 infection, which supported that AaCV1-QY2 played a dominant role in fungal host hypovirulence caused by the coinfection of mycoviruses. Nevertheless, it is still unknown whether AaMV1 plays a synergistic role or merely serves as an accompanying virus, and related experiments cannot be conducted to demonstrate this in this study because a single AaMV1-infected strain cannot survive in the subculture.

Members of the family *Chrysoviridae* have been identified to infect a wide array of fungal species and are considered to debilitate the virulence of their phytopathogenic fungal hosts (Moriyama et al., 2018; Kotta-Loizou et al., 2020; Casas et al., 2023). For example, the viruses *Colletotrichum fructicola* chrysovirus 1 (CfCV1), *Magnaporthe oryzae* chrysovirus 1-A (MoCV1-A), *F. oxysporum* f. sp. *dianthi* mycovirus 1 (FodV1), and BdCV1 induce hypovirulence in the their hosts (Moriyama et al., 2018; Wang et al., 2018; Zhai et al., 2018; Torres-Trenas et al., 2019). Also, in this study, AaCV1-QY2 infection reduced the synthesis of AOH toxins in the *A. alternata* host and alleviated the symptoms caused by host fungi on apple fruits and Begonia leaves. However, it has been reported that AaCV1-N18 isolated from *A. alternata* causing a pear black spot endowed the host with enhanced pathogenicity by promoting host-specific AK toxin level (Okada et al., 2018) and AaCV1-AT derived from *Alternaria tenuissima* causing watermelon leaf blight provided enhanced sensitivity of the host fungus to difenoconazole or tebuconazole (Ma et al., 2020). These two viruses share the closest homology with AaCV1-QY2, but their infection behavior had completely different effects on host pathogenicity, except that the growth of their respective hosts was consistently reduced compared with AaCV1-QY2. To our knowledge, this is the first report of hypovirulence caused by *chrysovirus* infection in the pathogenic fungus *Alternaria* species.

Recently, an increasing number of ourmia-like viruses have been identified in different fungi, but most of them have little or no impact on the virulence (Illana et al., 2017; Wang et al., 2020; Zhou et al., 2020). So far, only one ourmia-like mycovirus FoOuLV1, has been reported to induce hypovirulence (Zhao et al., 2020). However, FoOuLV1 is unique among ourmia-like mycoviruses because there are two segments, L-segment and S-segment, which were harbored in the host strain HuN8. And, the S-segment associated with FoOuLV1 was proven to play an important role in its hypovirulence (Zhao et al., 2020). In this study, the results of morphological comparison and virulence assay showed that the extra presence of AaMV1 had no superimposed influence on morphological alterations and the level of hypovirulence compared with AaMV1 elimination strain, but the accumulation of nHST toxin AOH, as a virulence factor, was restored to a certain extent, and it is positively correlated with the aggravation of disease symptoms and human health risks (Meena et al., 2017). We speculate that this contradictory phenomenon is because the presence of AaMV1 not only affected the metabolic pathway of AOH toxin in the host, but could also regulate other physiological metabolic processes related to host virulence, such as the synthesis of other types of nHST and HST toxins that were not detected in this study due to some technical obstacles. Finally, due to the combined effects of various factors, there was no significant difference in reducing host pathogenicity between the host strains with both mycoviruses and single AaCV1-QY2 infection.

Coinfection of mycoviruses is rather common in plant pathogenic fungi. Generally, co-infected mycoviruses are reported to be dependently harbored, there is a close and complex interaction between mycoviruses, which may affect the pathogenicity and transmission efficiency of mycoviruses, especially during transmission through vegetatively incompatible fungi (Kashif et al., 2019; Thapa and Roossinck, 2019). For example, the horizontal transmission efficacy of the mycovirus HetPV15-pa1 to a pre-infected host was elevated from 0 to 50% by the presence of HetPV13-an1. Furthermore, Heterobasidion partitiviruses in recipient strains have extremely varied impacts on the transmission of new viruses, depending on specific virus combinations, they can synergistically enhance the overall transmission rate or antagonistically alter their transmission (Kashif et al., 2019). In this study, the coinfection of both viruses (AaCV1-QY2 and AaMV1) did not alter the virulence of *A. alternata* compared with single AaCV1-QY2 infection, but improved the horizontal transmission rate of AaCV1-QY2 from CY21 strain to the virus-free strains CY21-C2 and to the other *A. alternata* strains via hyphal anastomosis. This change in interspecific transmission ability is associated with the coinfection involving the virus AaMV1, coinfection perhaps inducing the suppression of fungal non-self-recognition and RNA silencing are two most common protective mechanisms against virus infection and transmission. This hypothesis was confirmed by the study that hypovirulence-associated *S. sclerotiorum* mycoreovirus 4 (SsMYR4) suppresses non-self-recognition of the fungal host and facilitate coinfection through the horizontal transmission of mycoviruses between different strains (Wu et al., 2017). However, if there is more accurate information about vegetative compatibility between QY21 or QY21-C1 and the other 99 allogeneic virus-free strains, it would offer further insights into the impact of coinfection on the horizontal transmission of AaCV1-QY2. Taken together, the interaction between co-infected viruses and their hosts is multifaceted, their underlying mechanism remains to be elucidated, which depends on the property of the fungal host, the type of mycoviruses, and the interactions between the co-infected viruses themselves.

In recent studies, more and more hypovirulent mycoviruses have been identified and explored as potential biocontrol agents against fungal diseases. Nonetheless, with the exception of the two mycoviruses CHV1 and SsHADV-1, there are few attempts to use mycoviruses to control diseases in the field. So far, many mycoviruses have been identified from the genus *Alternaria*; but, to our knowledge, only seven mycoviruses have been identified in *A. alternata*. Furthermore, among them, only AaHV1 is considered to authentically induce hypovirulence. In this study, the coinfection of AaCV1-QY2 or AaCV1-QY2 + AaMV1 was not only able to forcibly induce hypovirulence in *A. alternata*, but also exhibited excellent horizontal transmission ability, especially with AaCV1-QY2 + AaMV1 infection. We also determined that these two mycoviruses could not infect Begonia leaves, so they would not pose a safety risk to plant health. These are considered to be important conditions and advantages for the successful application of mycoviruses to the control of fungal plant disease.

## Conclusion

We have characterized two mycoviruses (AaCV1-QY2 and AaMV1) related to members of the genera *Betachrysovirus* and *Megoulvirus*, respectively, from a phytopathogenic fungus, *A. alternata* f. sp. *mali* strain QY21. AaCV1-QY2 plays a dominant role in causing morphological alterations and hypovirulent phenotype. This is the first report of the phenomenon of hypovirulence conferred in *A. alternata* by the coinfection of two mycoviruses. Furthermore, AaMV1 harbored in *A. alternata* facilitates the horizontal transmission ability of the mycovirus AaCV1-QY2 and mediate the accumulation of the mycotoxin AHO. Therefore, these mycoviruses can be considered as potential biological control agents to control *Alternaria* blotch disease. However, further studies are needed to determine the role of each mycovirus in viral transmission, pathogenic behavior, physiological changes, and their interactions in influencing the biology and adaptability of *A. alternata* host strains.

## Data Availability Statement

The datasets presented in this study can be found in online repositories. The names of the repository/repositories and accession number(s) can be found below: https://www.ncbi.nlm.nih.gov/genbank/, MK672910; https://www.ncbi.nlm.nih.gov/genbank/, MK672913; https://www.ncbi.nlm.nih.gov/genbank/, MK672912; https://www.ncbi.nlm.nih.gov/genbank/, MK672911; https://www.ncbi.nlm.nih.gov/genbank/, MK836314; https://www.ncbi.nlm.nih.gov/genbank/, MW492 539; and https://www.ncbi.nlm.nih.gov/, PRJNA832420.

## Author Contributions

YW and KC designed the study and revised this manuscript. BL analyzed the data and wrote the manuscript. YC, JZ, PD, and ZJ performed the experimental work. XM, TH, and SW helped to revise this manuscript. All authors approved the final manuscript.

## Conflict of Interest

The authors declare that the research was conducted in the absence of any commercial or financial relationships that could be construed as a potential conflict of interest.

## Publisher’s Note

All claims expressed in this article are solely those of the authors and do not necessarily represent those of their affiliated organizations, or those of the publisher, the editors and the reviewers. Any product that may be evaluated in this article, or claim that may be made by its manufacturer, is not guaranteed or endorsed by the publisher.

## Footnotes

1
http://blast.ncbi.nlm.nih.gov/
