# Supplementary material for: Coinfection of Two Mycoviruses Confers Hypovirulence and Reduces the Production of Mycotoxin Alternariol in Alternaria alternata f. sp. mali
Source: Front Microbiol. 2022 Jun 9;13:910712. doi: 10.3389/fmicb.2022.910712 (PMC9218907; doi:10.3389/fmicb.2022.910712)
Supplement: Supplementary file 1 [file Table_1.DOCX]

# Supplementary Material

**
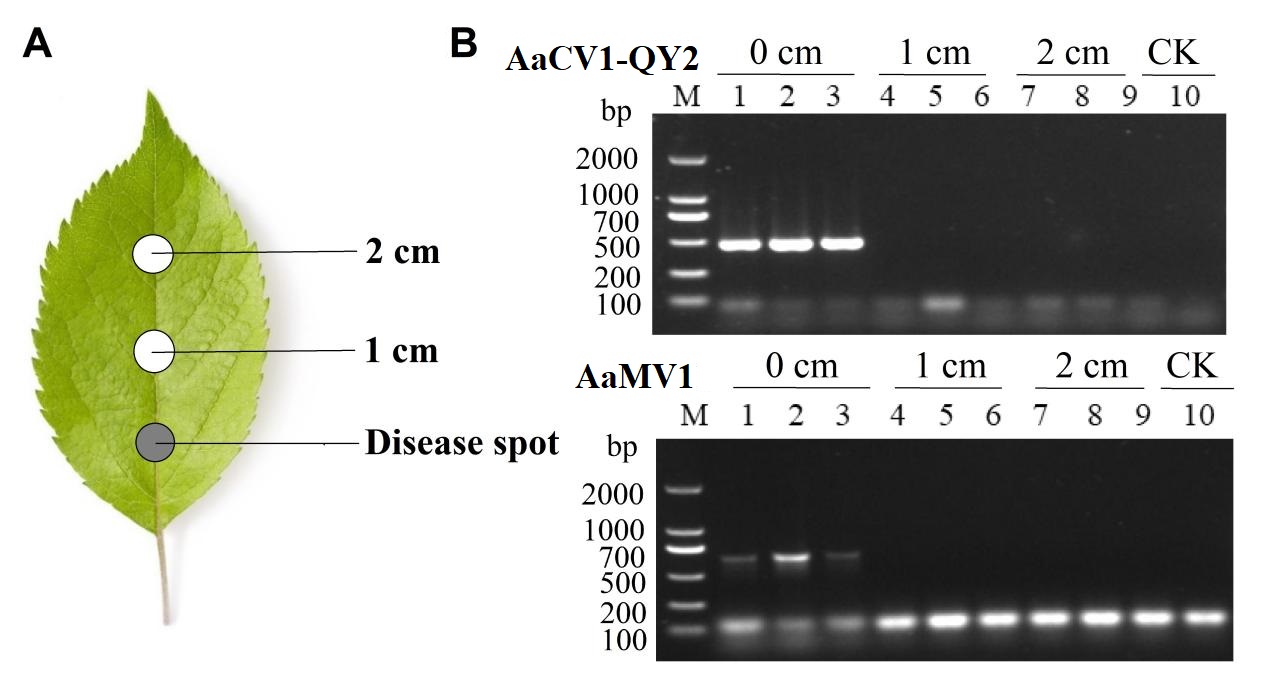
FIGURE S1 |** Detection of viruses AaCV1-QY2 or AaMV1 in Begonia leaf obtained from QY21 strain inoculation experiments. After 1 week of inoculation, leaf tissues were taken from three locations (0 cm, 1 cm, 2 cm away from the inoculation location) along the midvein for total RNA extraction. CK: The leaves of Begonia inoculated with virus-free strain QY21-C2 were used as samples to extract total RNA.


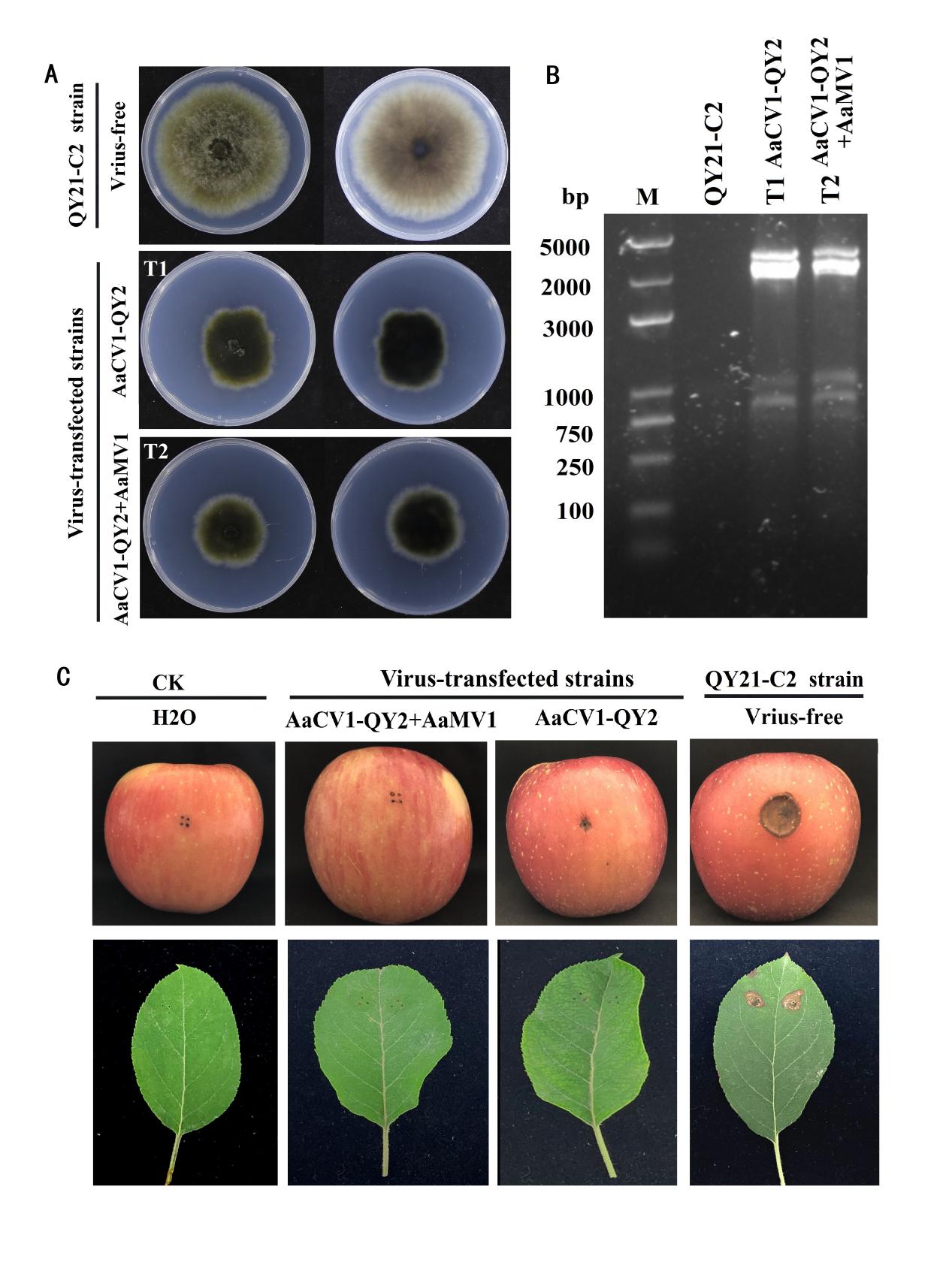


**FIGURE S2 |** The biological characteristics and dsRNA pattern of virus-transfected *Alternaria alternata* strains. Virus-transfected strains came from the fifth generation of QY21-C2 strain that was used as recipient in co-culture experiment. Virus-transfected strain T1 carries a virus AaCV1-QY2, and QY21-C1 as donors in co-culture experiment; virus-transfected strain T2 carries two viruses AaCV1-QY2 and AaMV1, and strain QY21 in co-culture experiment. **(A)** Colony morphology of strain T1 and T2 (PDA, 4 days, 25˚C). **(B)** Pathogenicity of strain T1 and T2 on the apple fruits or detached Begonia leaves (5 d post-inoculation at 25˚C). CK, blank control. **(C)** Agarose gel electrophoretic analysis of the dsRNA extracted from strains QY21-C1 (lane 1), T1 (lane 2) and T2 (lane 3). The dsRNA was treated with DNase I. M: Molecular weight marker.


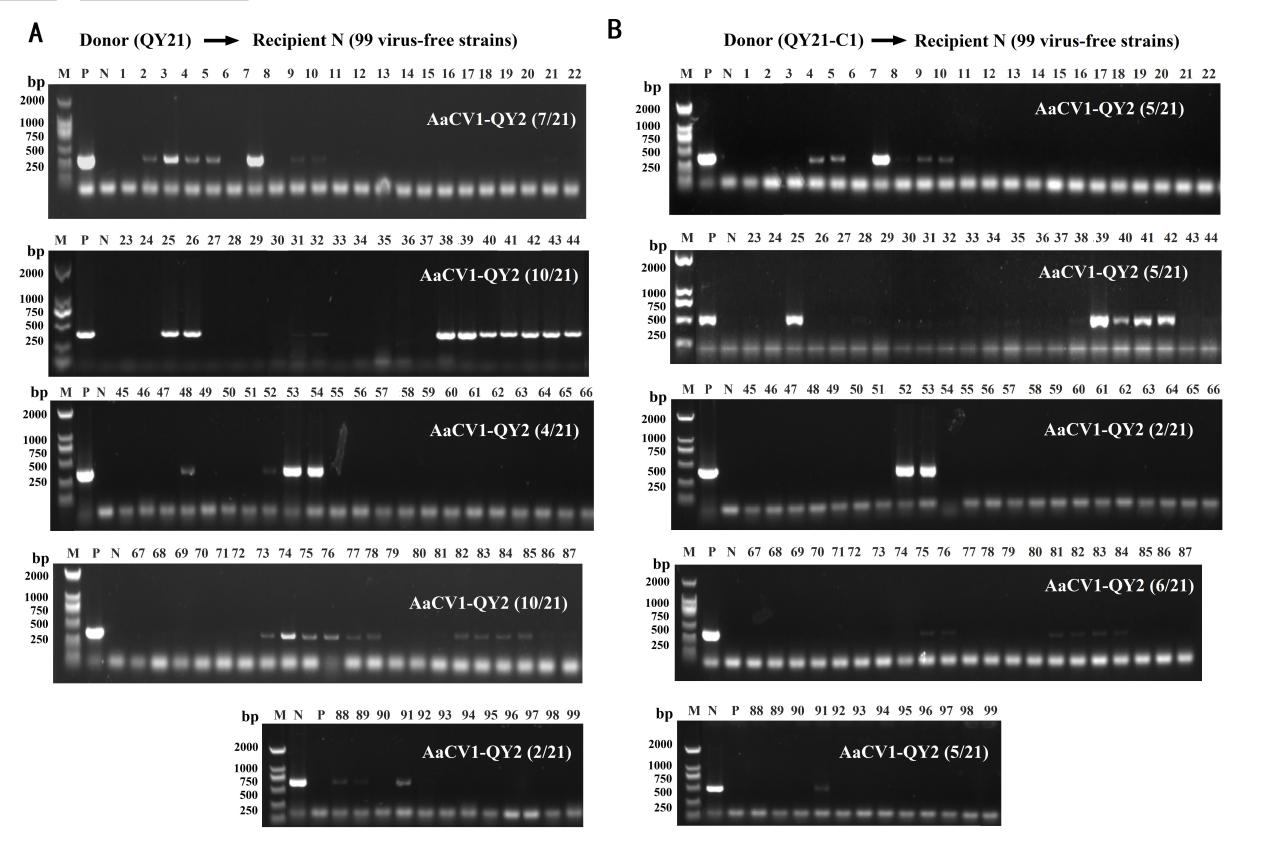


**FIGURE S3 |** The efficiency of AaCV1-QY2 and AaMV1 horizontal transmission via hyphal anastomosis in *Alternaria alternata*. The virus-infected strains QY21 (left) and QY21-C1 (right) as donors and other 99 allogenetic virus-free strains collected from different orchards as recipients. **(A)** Detection of AaCV1-QY2 in recipient N strains co-cultured with AaCV1-QY2+AaMV1-infected strain QY21. **(B)** Detection of AaCV1-QY2 in recipient N strains co-cultured with AaCV1-QY2-infected strain QY21-C1. The numbers in the electrophoresis chart indicate the number of samples with AaCV1-QY2 detected in each batch of receptors. P: Positive control; N: Negative control; M: Molecular weight marker.

**TABLE S1 |** Information of primers used in this study.

| **Primer name** | **Primer sequence(5'-3')** | **Size of product (bp)** |
| --- | --- | --- |
| **For detecting virus AaCV1-QY2** | | |
| AaCV1-QY2f | ATCTCAAGGTACCGGAGTCA | 450 |
| AaCV1-QY2r | GCTGCTTAAACATCACCTGCA |  |
| **For detecting virus AaMV1** | | |
| AaMV1f | TTTGTAGTGGACTTATTGAACGTCG | 642 |
| AaMV1r | CAGTTGCTGATTCGTAGTCTCCAGA |  |
| **For detecting fungi *Alternaria alternata*** | | |
| TEF-1αf | CCGAACTCGGTAAGGG | 1509 |
| TEF-1αr | CATACAGAGCGATGTGATAGAC |  |
| ***To obtain the 3’-end regions of each dsRNA segment*** | | |
| ***3’-***segment 1 | GATTACGCCAAGCTTTTCTCGCGGTACTTCTGGAAGATGGAGCCTGG | |
| ***3’-***segment 2 | GATTACGCCAAGCTTCCATGCCTGCTCGCAGCTTGCTGCGAG | |
| ***3’-***segment 3 | GATTACGCCAAGCTTGCTGGCCGCGAGTAGACATGGGTTCGG | |
| ***3’-***segment 4 | GATTACGCCAAGCTTGGACATGGGTTCGAGAGGGTTTTTGGTT | |
| ***3’-***segment 5 | GATTACGCCAAGCTTCTGGAACAAGAGGCAAGCTGCCCC | |
| ***3’-***segment 6 | GATTACGCCAAGCTTGGTCGTGCCGTCCCAAAGGTTACAGG | |
| ***To obtain the 5’-end regions of each dsRNA segment*** | | |
| ***5’-***segment 1 | AAGCTTGGCGTAATCACCTGCCTGAACCACTCGTAAGCGCCGTC | |
| ***5’-***segment 2 | AAGCTTGGCGTAATCAATGCCGCACGCAGCCAGCTG | |
| ***5’-***segment 3 | AAGCTTGGCGTAATCGCCGCCGACAGCCAGCTGCCGGAAGC | |
| ***5’-***segment 4 | AAGCTTGGCGTAATCGCACGCAGCCAGCTGCGCGAAGC | |
| ***5’-***segment 5 | AAGCTTGGCGTAATCGGAGCCACCGCAGCTCGCGGTTCCAC | |
| ***5’-***segment 6 | AAGCTTGGCGTAATCCCTGTAACCTTTGGGACGGCACGACC | |
